# Increased Expression of the dsRNA-Activated Protein Kinase PKR in Breast Cancer Promotes Sensitivity to Doxorubicin

**DOI:** 10.1371/journal.pone.0046040

**Published:** 2012-09-24

**Authors:** Richard L. Bennett, Aubrey L. Carruthers, Teng Hui, Krystal R. Kerney, Xiangfei Liu, W. Stratford May

**Affiliations:** Department of Medicine, Division of Hematology and Oncology and the University of Florida Shands Cancer Center, University of Florida, Gainesville, Florida, United States of America; Vanderbilt University, United States of America

## Abstract

It has been reported that the expression and activity of the interferon-inducible, dsRNA-dependent protein kinase, PKR, is increased in mammary carcinoma cell lines and primary tumor samples. To extend these findings and determine how PKR signaling may affect breast cancer cell sensitivity to chemotherapy, we measured PKR expression by immunohistochemical staining of 538 cases of primary breast cancer and normal tissues. Significantly, PKR expression was elevated in ductal, lobular and squamous cell carcinomas or lymph node metastases but not in either benign tumor specimens or cases of inflammation compared to normal tissues. Furthermore, PKR expression was increased in precancerous stages of mammary cell hyperplasia and dysplasia compared to normal tissues, indicating that PKR expression may be upregulated by the process of tumorigenesis. To test the function of PKR in breast cancer, we generated MCF7, T-47D and MDA-MB-231 breast cancer cell lines with significantly reduced PKR expression by siRNA knockdown. Importantly, while knockdown of PKR expression had no effect on cell proliferation under normal growth conditions, MCF7, T-47D or MDA-MB-231 cells with reduced PKR expression or treated with a small molecule PKR inhibitor were significantly less sensitive to doxorubicin or H_2_O_2_-induced toxicity compared to control cells. In addition, the rate of eIF2α phosphorylation following treatment with doxorubicin was delayed in breast cancer cell lines with decreased PKR expression. Significantly, treatment of breast cancer lines with reduced PKR expression with either interferon-α, which increases PKR expression, or salubrinal, which increases eIF2α phosphorylation, restored doxorubicin sensitivity to normal levels. Taken together these results indicate that increased PKR expression in primary breast cancer tissues may serve as a biomarker for response to doxorubicin-containing chemotherapy and that future therapeutic approaches to promote PKR expression/activation and eIF2α phosphorylation may be beneficial for the treatment of breast cancer.

## Introduction

The interferon (IFN)-inducible, double-stranded RNA-activated protein kinase, PKR, is present in most mammalian cells in a latent or inactive state. It has been well studied as an important component of the IFN-stimulated host antiviral defense mechanism. In this context, PKR is induced by IFN and activated by viral double-stranded RNA to catalyze phosphorylation of eIF2α resulting in global protein synthesis inhibition and initiation of apoptosis. Significantly, our laboratory and others have determined that PKR may be activated by a variety of cellular stresses such as hematopoietic growth factor starvation, inflammatory cytokines and chemotherapy agent treatment. [1], [2], [3] In addition to an inhibitor of translation, PKR has been reported to have an important role in signaling pathways such as NF-κB, p53 and STAT1 that regulate proliferation and apoptosis during cellular stress. [4], [5], [6], [7], [8], [9], [10], [11], [12], [13], [14], [15] Thus, PKR may serve as a guardian of the cell that facilitates the response to diverse stress stimuli.

The role of PKR in tumorigenesis is not well characterized. In general, PKR is considered to have a tumor suppressor function since increased PKR activity has been correlated with decreased cell proliferation and an anti-tumor activity [16], [17], [18]. In support of this, mutant forms of PKR and PKR’s downstream target, eIF2α, as well as inhibitors of PKR such as TRBP or p58 can induce transformation of cells. [19], [20] Furthermore, the loss of PKR catalytic activity has been observed in B-cell chronic lymphocytic leukemia patient samples, and an inactivating point mutant in PKR’s dsRNA binding has been detected in a small set of patients with acute lymphoblastic leukemia of T-cell lineage. [21], [22] The PKR gene is located on 2p21-22, a chromosomal region that has been associated with large cell lymphoma, myelodysplastic syndrome and acute myelogenous leukemia. [23], [24], [25], [26], [27] In addition, the PKR gene is transcriptionally regulated by IFNs α and γ via IRF-1, and down regulation of PKR has been shown to occur in 5q- associated leukemias that delete the IRF-1 gene. [28], [29], [30], [31] Significantly, it has been recently reported that primary non-small cell lung cancer (NSCLC) samples have decreased PKR expression compared to normal bronchial epithelium. [32] Furthermore, loss of PKR expression correlates with a more aggressive behavior while high PKR expression predicts a subgroup of NSCLC patients with a favorable outcome. [32] Collectively, these findings suggest that PKR may play an important role in tumor suppression and that inhibition of PKR activity is associated with tumorigenesis.

As an initiator of apoptosis in response to cellular stress, PKR may mediate the sensitivity of cancer cells to chemotherapy. For example, PKR is activated by the anthracycline doxorubicin (DOX), a commonly used treatment for a wide range of cancers. [33] Following DOX application, PKR has been reported to induce apoptosis of cancer cell lines by mechanisms dependent on eIF2α phosphorylation, p53 phosphorylation and JNK activation. [33], [34] Importantly, in a mouse xenograft model, colon cancer cells with reduced PKR expression more rapidly established solid tumors that were resistant to DOX or etoposide treatment compared to control cells. [35] In addition, it has been reported that PKR promotes 5-Fluorouracil (5-FU)- induced apoptosis by a mechanism dependent on eIF2α phosphorylation. [36] Significantly, knockdown of PKR expression in colon and breast cancer cell lines resulted in a decreased sensitivity to 5-FU and eliminated the ability of IFNα to improve 5-FU cytotoxicity. [36].

To better understand the role of PKR signaling in breast cancer cell proliferation and response to chemotherapy, we analyzed PKR expression and function in both primary breast cancer tissues and 3 common breast cancer cell lines. Previous work from other laboratories indicates PKR expression is elevated both in primary ductal carcinoma tissues compared to normal luminal ductal epithelial samples, and in breast cancer derived cell lines than nontransformed mammary epithelial cell lines. [37], [38], [39]. To extend these findings, we measured PKR expression by immunohistochemical (IHC) staining of primary breast cancer tissue microarrays containing 538 cases. Significantly, results indicate that PKR is elevated in invasive ductal, lobular and squamous cell carcinomas as well as in regional lymph node metastasis compared to normal breast tissue. Furthermore, PKR expression is increased in precancerous stages of mammary cell hyperplasia and dysplasia compared to normal tissues but not cases of breast tissue inflammation, indicating that PKR expression may be upregulated during tumorigenesis. In addition, we investigated the response to DOX in breast cancer cell lines MCF7, T-47D or MDA-MB-231 with significantly reduced PKR expression by siRNA knockdown. Importantly, breast cancer cell lines with reduced PKR expression or treated with a PKR inhibitor are less sensitive to DOX or H_2_O_2_ -mediated cytotoxicity compared to control cells. Furthermore, following treatment with DOX, breast cancer cell lines with reduced PKR expression have a decreased rate of eIF2α phosphorylation compared to control cells. In addition, treatment of MCF7, T-47D or MDA-MB-231 cells with IFNα, to increase PKR expression, or with salubrinal, to increase phosphorylated eIF2α, increases DOX cytotoxicity and restores DOX sensitivity in cells with reduced PKR expression to that of control cells. Taken together these results suggest that increased activation of PKR-eIF2α signaling observed in breast cancer specimens may contribute to the therapeutic index of DOX-containing chemotherapy. Thus, PKR expression may serve as a biomarker for DOX sensitivity and strategies to increase PKR-eIF2α signaling may be therapeutically useful for breast cancer in the future.

## Results

### PKR Expression is Increased in Primary Breast Cancer Tissues

To investigate the clinical relevance of PKR expression in breast cancer, we measured PKR level by high throughput immunohistochemical (IHC) analysis of tissue microarrays containing 538 primary samples. The arrays consisted of 154 normal or cancer adjacent normal, 243 malignant, 47 lymph node metastasis, 37 benign fibroadenoma, 31 hyperplasia, 10 dysplasia, and 16 inflammation cases. Malignant cases consisted of 167 invasive ductal carcinomas, 34 invasive lobular carcinomas, 2 carcinosarcomas, 8 cystosarcoma phyllodes, 4 lobular carcinoma in situ, 9 medullary carcinomas, 8 mucinous adenocarcinomas, 3 mucous carcinomas, 4 Paget’s disease, and 4 squamous cell carcinomas. IHC staining for PKR was scored on a scale of 0 (no staining) to 9 (strong, 100% staining). The number of tissue cores examined per case varied from 1 to 3, and PKR staining scores for cases with duplicate or triplicate cores were averaged.

**Figure 1:**
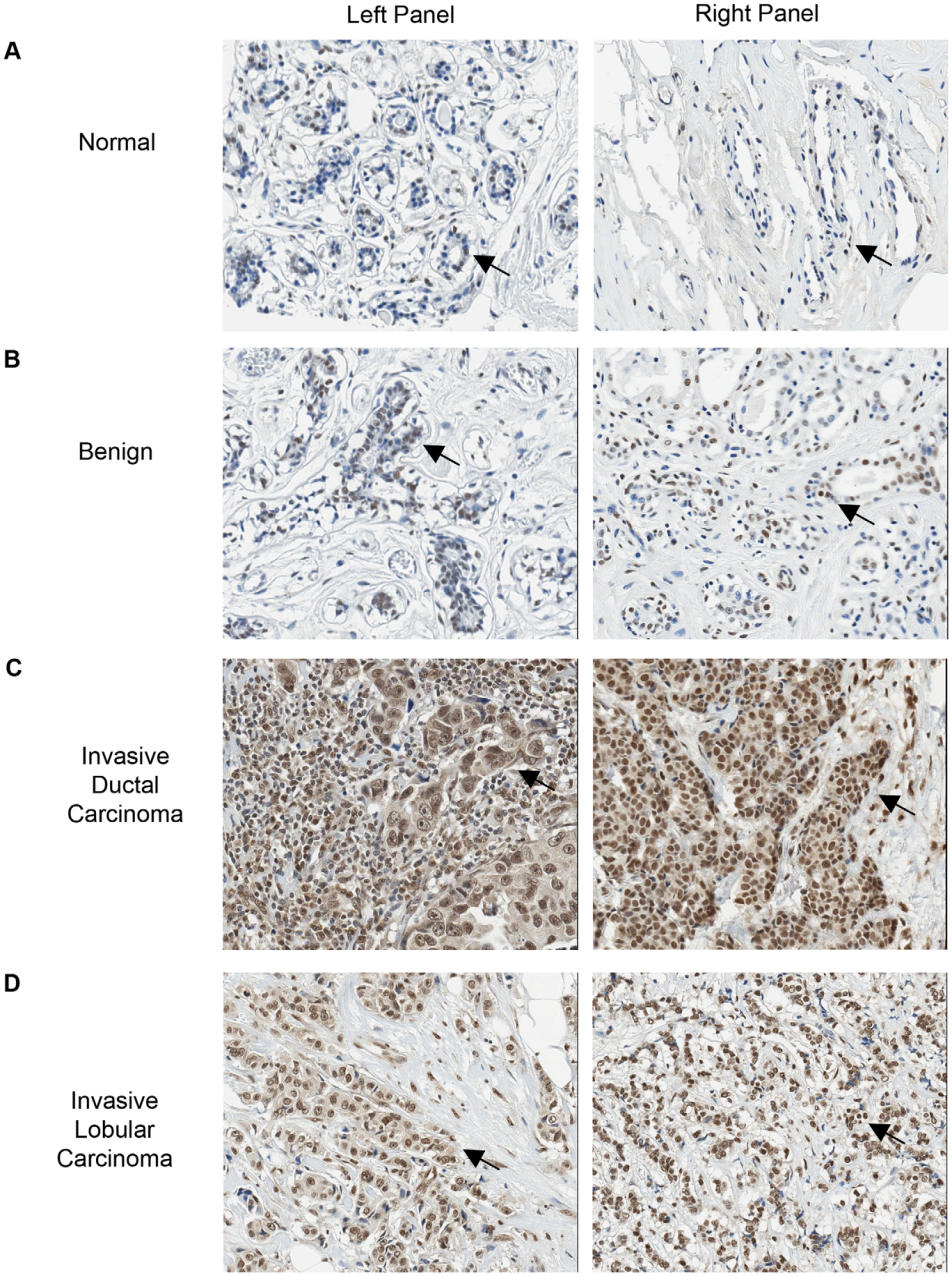
PKR is significantly elevated in primary breast cancer tissues compared to normal or benign tissues. Representative results from IHC staining (20× magnification shown). PKR is stained brown and nuclei are stained blue. Arrows highlight positively stained cells. **A**. Normal breast tissues. Left panel: 44 year old female; Right panel: 35 year old female. **B**. Benign: Left panel: 44 year old female, adenosis; Right: 35 year old female, blunt duct adenosis. **C**. Invasive ductal carcinomas: Left panel: 57 year old female, IDC not otherwise specified, Stage 3; Right Panel: 45 year old female, grade 1 T4N1M0. **D**. Invasive lobular carcinomas: Left panel: 35 year old female; Right panel: 38 year old female.

Significantly, IHC staining for PKR was increased in malignant compared to normal mammary epithelial tissue (mean score 6.892 vs. 4.569, P<0.0001; Figure 1 and Table 1). Furthermore, increased PKR expression compared to normal tissues was statistically significant for the more aggressive tumors including invasive ductal and lobular carcinomas as well as squamous cell carcinomas (Figure 1 and Table 1). All grades (1 to 3) of invasive ductal carcinoma displayed uniformly elevated PKR expression compared to normal tissues. However, no significant difference between the tumor grades (1 to 3) of invasive ductal carcinomas was observed (Table 1). Moreover, PKR was increased in lymph node metastasis compared to normal tissues (6.887 vs. 4.569, P<0.0001; Table 1). In contrast, no significant difference in PKR levels could be observed between other types of breast cancer examined or between benign vs. normal specimens (Table 1).

**Table 1:** Immunohistochemical analysis of PKR expression in normal and breast cancer TMA specimens.

Pathology diagnosis	No. of cases	Mean PKR score	*P*
**Normal**	154	4.569±0.2465	reference
Normal	93	3.675±0.2700	
Cancer adjacent normal	61	5.932±0.4119	
**Malignant**	243	6.892±0.1684	<0.0001
Ductal carcinomas	167	7.283±0.1854	<0.0001
Grade 1	19	7.474±0.6247	<0.0001
Grade 2	87	7.339±0.2551	<0.0001
Grade 3	26	8.244±0.3477	<0.0001
Lobular carcinomas	34	7.735±0.2804	<0.0001
Squamous cell carcinoma	4	8.250±0.7500	0.0179
Others: Carcinosarcoma (N = 2), Cystosarcoma phyllodes (N = 8), Lobular carcinoma In Situ(N = 4), Medullary carcinoma (N = 9), Mucinous adenocarcinoma (N = 8), Mucous carcinoma(N = 3), Paget’s disease (N = 4)	38	4.325±0.4789	0.6576
**Lymph node metastasis**	47	6.887±0.3530	<0.0001
**Benign**	37	4.878±0.4186	0.5697
**Hyperplasia**	31	6.613±0.3661	0.0005
**Dysplasia**	10	8.200±0.5281	0.0003
**Inflammation**	16	4.146±0.4868	0.5892

**Figure 2:**
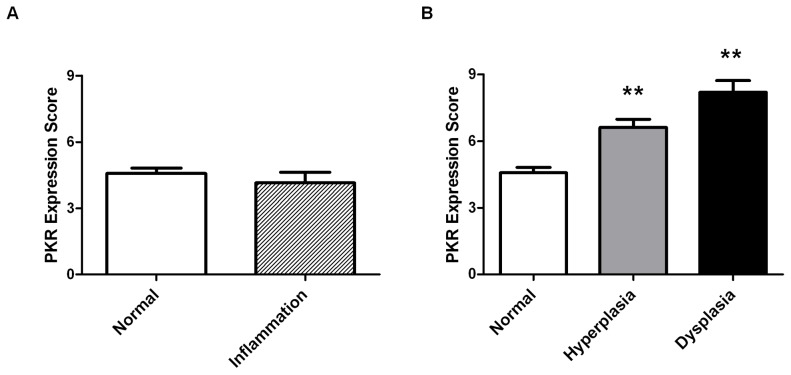
Increased PKR expression in breast tissue specimens coincides with transformation but not inflammation. **A**. No significant difference in PKR expression is observed between cases of inflammation (n = 16) and normal (n = 154) breast tissues. **B**. Both hyperplasia (n = 31) and dysplasia (n = 10) precancerous tissue specimens display increased PKR expression compared to normal tissues (n = 154). Statistical significance was determined by t-test. ** Indicates p<0.01.

In addition, to assess the point during malignant transformation that PKR expression may be increased, we analyzed precancerous and inflammation tissue specimens. No significant difference in PKR expression was observed in breast inflammation (including cases of mastitis or chronic inflammation) compared to normal specimens (Table 1 and Figure 2A). In contrast, potentially precancerous tissues including hyperplasias and dysplasias demonstrated significantly elevated PKR expression compared to normal (Table 1 and Figure 2B). These results suggest that PKR expression may be increased during the process of malignant transformation by an unknown mechanism.

### PKR Expression in Breast Cancer Cell Lines is Required for Cell Invasion

**Figure 3:**
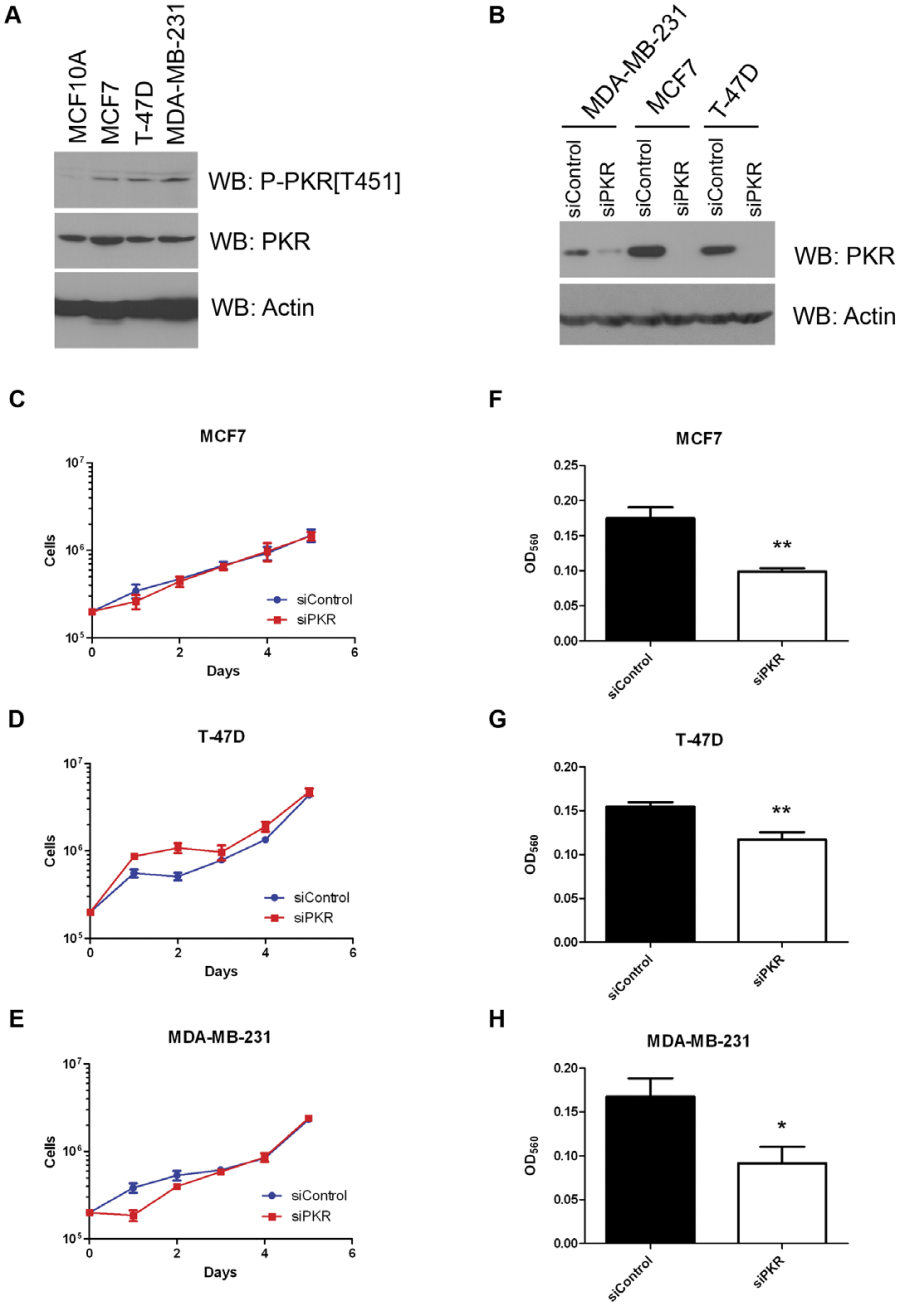
Increased levels of phosphorylated PKR are present in breast cancer cell lines. A . Western blotting for phospho-T451-PKR in breast cancer (MCF7, T-47D, MDA-MB-231) and “normal” breast epithelial (MCF10A) cell lines demonstrates increased basal levels of “activated” PKR present in breast cancer cell lines compared to normal breast cells. **B**. Western blots showing that PKR level is significantly decreased in breast cancer cell lines that stably express siRNA specific to PKR (siPKR) compared to control siRNA (siControl). **C.–E**. Breast cancer cell lines with decreased levels of PKR proliferate at the same rate as control cells. Experiments were done in triplicate, mean ± SD is shown. **F. – H**. Breast cancer cell lines with reduced PKR expression by siRNA knockdown display reduced level of cell invasion compared to control cells. Briefly 20,000 cells were added to the upper chamber of a transwell insert in serum free medium while medium containing 10% FBS was placed in the lower chamber. After 24 hours, invading cells in the lower chamber were stained and OD_560_ measured. Experiments were repeated four times and mean ± SD was graphed. Statistical significance was determined by t-test. * Indicates p<0.05, ** indicates p<0.01.

To determine the significance of increased PKR expression on breast cancer cell proliferation and sensitivity to chemotherapy agents, we tested the role of PKR in the breast cancer cell lines MCF7, T-47D and MDA-MB-231. Interestingly, basal PKR phosphorylation at threonine 451, a critical site of PKR autophosphorylation, was dramatically higher in breast cancer cell lines MCF7, T-47D and MDA-MB-231 compared to the “nontransformed” mammary epithelial cell line MCF10A (Figure 3A). To determine whether PKR level and activity may be required for proliferation or tumor response to chemotherapy agents, we employed a siRNA strategy to knockdown PKR by >90% in breast cell lines MCF7 and T-47D and >50% in MDA-MB-231 (Figure 3B). Significantly, breast cancer cell lines with reduced PKR expression did not display a defect in cell proliferation under standard growth conditions (Figure 3C–E).

Since our laboratory and others have previously found that PKR is required for the response to serum withdrawal and growth factor starvation, we tested whether PKR expression effected breast cancer cell invasion. [1], [3] Briefly, cells were seeded into a Boyden chamber in serum-free medium while serum-containing medium was placed in the lower chamber. Cell invasion through the extracellular matrix was scored after 24 hours. Significantly, MCF7, T-47D and MDA-MB-231 cells with reduced PKR expression displayed reduced cell invasion (Figure 3F- H). These results may indicate that the PKR-dependent response to growth factor starvation is required for cell invasion and that increased PKR expression in breast cancer cells may promote cell invasion.

### Breast Cancer Cells with Reduced PKR Expression are Less Sensitive to Doxorubicin

**Figure 4:**
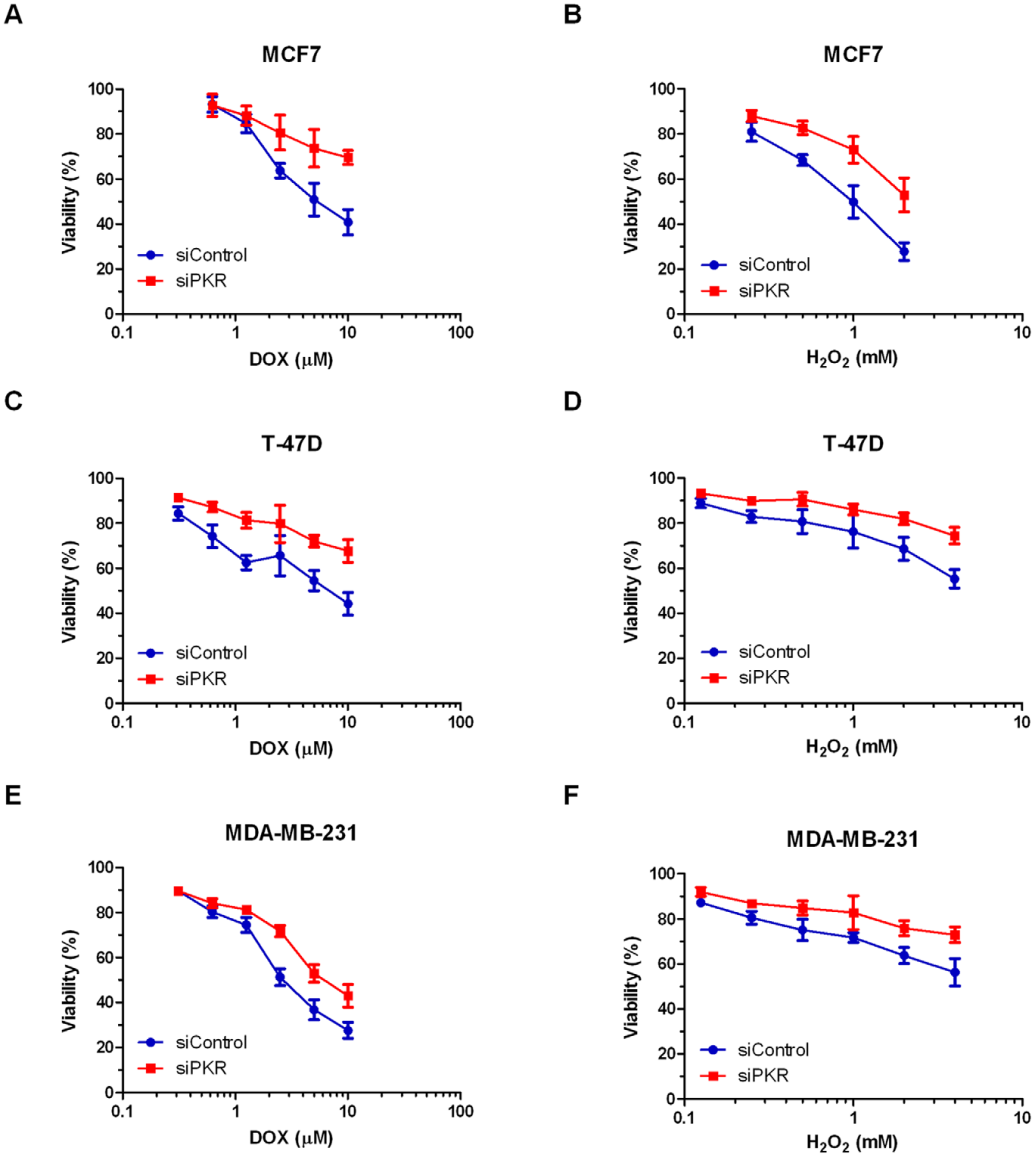
Breast cancer cell lines with reduced PKR are less sensitive to doxorubicin or H_2_O_2_. Breast cancer cell lines stably expressing either siRNA to PKR (siPKR) or control siRNA (siControl) were treated with increasing concentrations of doxorubicin (DOX) or H_2_O_2_ for 48 hours. Viability was measured by Trypan blue dye exclusion assay. Experiments were repeated in triplicate and mean ± SD graphed. **A**. MCF7 cell lines treated with DOX. **B**. MCF7 cell lines treated with H_2_O_2_. **C**. T-47D cell lines treated with DOX. **D**. T-47D cell lines treated with H_2_O_2_. **E**. MDA-MB-231 cell lines treated with DOX. **F**. MDA-MB-231 cell lines treated with H_2_O_2_.

To determine the role of PKR on breast cancer cell response to chemotherapy agents, MCF7, T-47D and MDA-MB-231 cells expressing either siRNA to PKR or control siRNA were treated with doxorubicin (DOX) and viability was measured by Trypan blue dye exclusion assay. Importantly, breast cancer cell lines with reduced PKR expression are significantly less sensitive to increasing concentrations of DOX than control cells (Figure 4A–C). Significantly, following 48 hours treatment with 10 µM DOX, MCF7 or T-47D cells with reduced PKR display an almost 25% increase in survival compared to cells expressing a control siRNA (Figure 4A and C). Similarly, MDA-MB-231 cells with reduced PKR display an almost 15% increase in cell survival compared to control cells (Figure 4E). In addition, following treatment of MCF7 cells with 5 µM DOX for various times, cells with reduced PKR display a reduced rate of cell death compared to control cells (Figure S1A). In addition, to confirm that DOX-treated breast cancer cell lines die by apoptosis, we performed a TUNEL assay. Importantly, DOX treatment induces DNA fragmentation detected by TUNEL assay in breast cancer cell lines and following 24 hours treatment with 5 µM doxorubicin, MCF7 and T-47D cells with reduced PKR exhibit a reduction in TUNEL positive cells compared to cells expressing a control siRNA (Figure S1B). Taken together these results indicate that PKR expression promotes sensitivity to DOX-induced apoptosis in breast cancer cells.

Since one consequence of DOX treatment is increased ROS that may mediate cytotoxicity, we tested the sensitivity of breast cancer cell lines with reduced PKR to H_2_O_2_ treatment. Significantly, following 48 hours treatment with increasing concentrations of H_2_O_2_, MCF7, T-47D and MDA-MB-231 cells expressing PKR siRNA have a reduced rate of cell death compared to control siRNA cells (Figure 4B, D, and F). Importantly, following 48 hours treatment with 2 mM H_2_O_2_, MCF7 cells with reduced PKR expression display an ∼25% increase in viability compared to control cells (Figure 4B). Furthermore, following 48 hours treatment with 4 mM H_2_O_2_, T-47D or MDA-MB-231 cells with reduced PKR expression display an ∼20% increase in viability compared to control cells (Figure 4D and 4F).

In addition, we tested whether PKR level may effect breast cancer cell line sensitivity to another standard and potent chemotherapy for breast cancer, paclitaxel. Interestingly, MCF7 cells with reduced levels of PKR display approximately the same sensitivity to paclitaxel as control cells (Figure S1C). Furthermore, co-treatment of MCF7 cells with the combination of DOX and paclitaxel, demonstrates that PKR has no effect on the synergy between these two compounds, since any difference in viability can be attributed to the reduced sensitivity to DOX previously observed (Figure S1D). Thus, PKR may be important for the response to DOX and ROS but not involved in the response to microtubule stress.

### PKR Activity and Increased Expression Promote Doxorubicin Sensitivity

**Figure 5:**
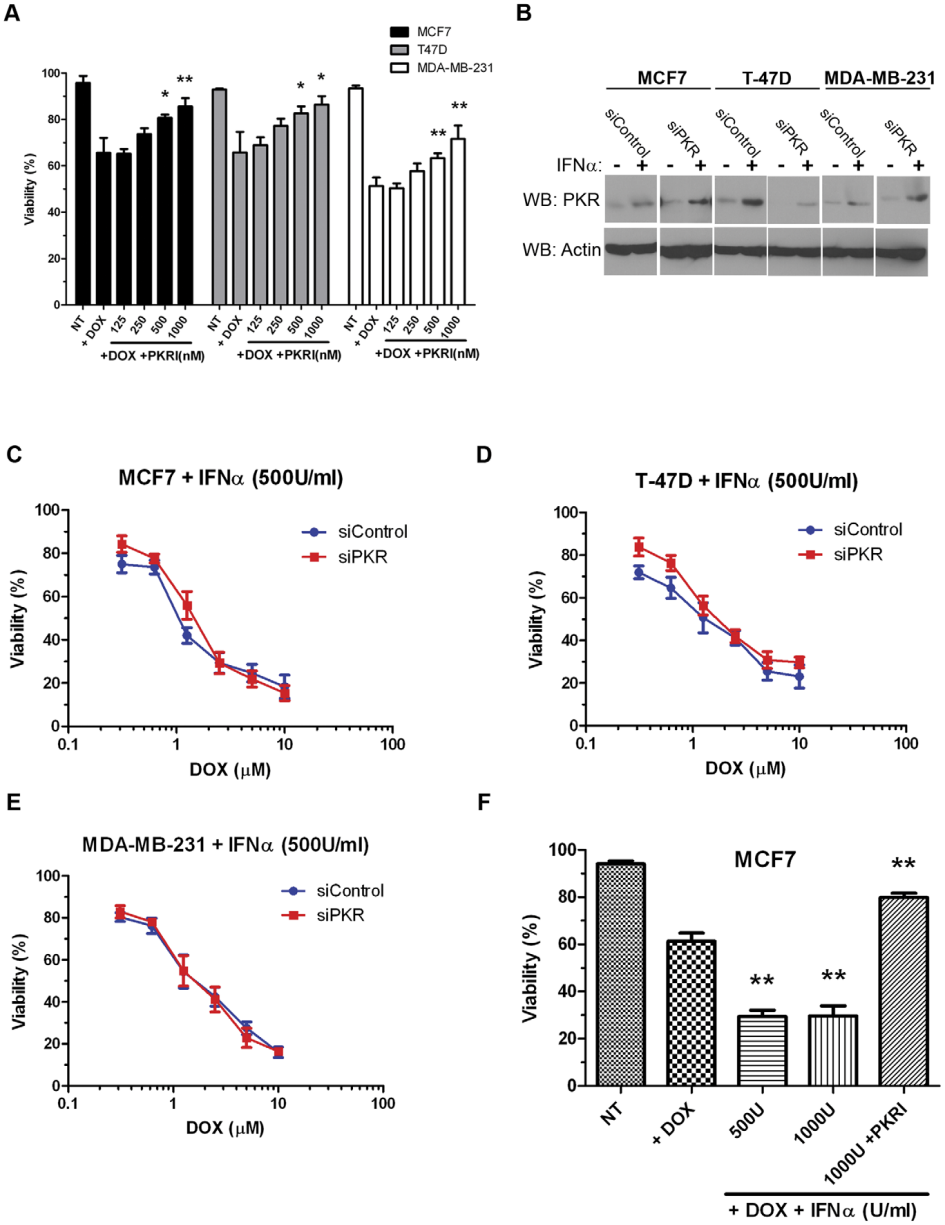
PKR activity and expression promotes sensitivity to doxorubicin. A . Breast cancer cell lines were treated with either 2.5 µM doxorubicin (DOX) or co-treated with 2.5 µM DOX and increasing concentrations of PKR inhibitor (PKRI) for 48 hours. Cell viability was measured by Trypan blue dye exclusion assay. Experiments were repeated in triplicate and mean ± SD graphed. Statistical significance was determined by t-test. * Indicates p<0.05, ** indicates p<0.01. **B**. Western blotting demonstrates increased PKR expression following treatment of breast cancer cell lines with 500 Units/ml Interferon-α (IFNα) for 48 hours in both control siRNA (siControl) and PKR siRNA (siPKR) expressing cells. **C.–E**. IFN-induced PKR expression increases breast cancer cell line sensitivity to doxorubicin. Breast cancer cell lines stably expressing either siRNA to PKR (siPKR) or control siRNA (siControl) were treated with increasing concentrations of doxorubicin (DOX) in medium containing 500 U/ml IFNα for 48 hours. Viability was measured by Trypan blue dye exclusion assay. Experiments were repeated in triplicate and mean ± SD graphed. **F**. PKR activity is required for IFN-induced sensitivity to DOX. MCF7 cells were treated for 48 hours either with 2.5 µM DOX and IFNα, or co-treated with DOX, IFNα and 1 µM PKRI. Cell death was measured by Trypan blue dye exclusion assay. Cell viability was measured by Trypan blue dye exclusion assay. Experiments were repeated in triplicate and mean ± SD graphed. Statistical significance was determined by t-test. ** Indicates p<0.01 compared to DOX treatment alone.

Since high levels of phospho-threonine 451 PKR are present in breast cancer cell lines that may indicate high levels of basal PKR activity, we tested whether specific inhibition of PKR activity could protect cells from DOX-induced cytotoxicity. Briefly, MCF7, T-47D or MBA-MD-231 cells were treated either with 2.5 µM DOX alone or with 2.5 µM DOX and increasing concentrations of a small molecule PKR inhibitor (PKRI) for 48 hours. Importantly, treatment with increasing concentrations of PKRI inhibited sensitivity to DOX for the three breast cancer cell lines tested. For instance, co-treatment with 2.5 µM DOX and 1 µM PKRI resulted in a ∼20% reduction in cytotoxicity compared to treatment with 2.5 µM DOX alone in all three cell lines (Figure 5A). These results indicate that PKR activity may facilitate full and potent sensitivity to doxorubicin.

Since, PKR is a well-characterized IFN-inducible gene, we next tested whether increasing the level of PKR by IFN treatment could affect sensitivity to DOX. Treatment of breast cancer cell lines with IFNα significantly increased the level of PKR as detected by western blotting (Figure 5B). Next, breast cancer cell lines were co-treated with both 500 Units/ml of IFNα and increasing DOX concentrations for 48 hours. Importantly, co-treatment with IFNα promoted a 2–5 fold increase in sensitivity of breast cancer cell lines to DOX (Figures 5C – E compared to Figures 4A, C and E). Furthermore, DOX sensitivity of cell lines expressing PKR siRNA was nearly restored to the level of control cells by co-treatment with IFNα (Figure 5 C – E). Importantly, the ability of IFNα to promote sensitivity to DOX was inhibited by co-treatment with PKRI (Figure 5F). These results illustrate that increased expression and activity of PKR may be critical for breast cancer cell sensitivity to doxorubicin.

### Phosphorylation of the PKR Target, eIF2α, Promotes Sensitivity to Doxorubicin

**Figure 6:**
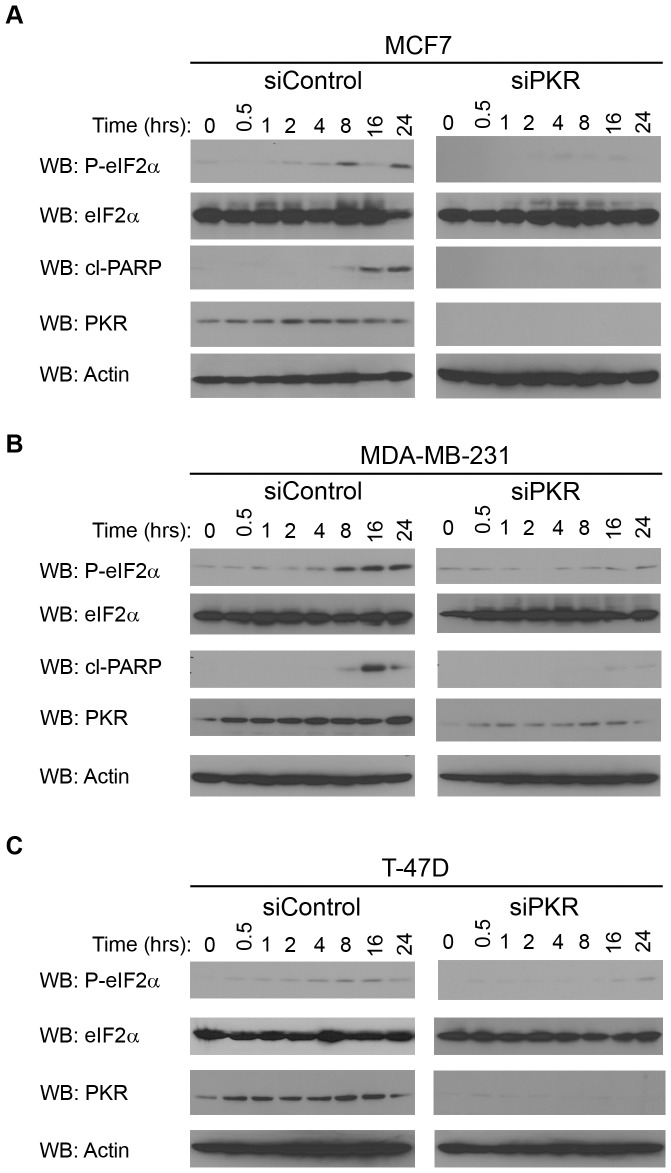
PKR expression is required for eIF2α phosphorylation following treatment of breast cancer cell lines with doxorubicin. Breast cancer lines with reduced PKR expression (siPKR) or control cells (siControl) were treated with 2.5 µM DOX for the indicated times. Following treatment, cells were collected and western blotting using antibody specific for phosphoserine-51 eIF2α (P-eIF2α), eIF2α, cleaved PARP (cl-PARP), PKR or actin was performed. A. Western blot of MCF7 cells following treatment with DOX indicates cells with reduced PKR have a reduced rate of eIF2α phosphorylation and PARP cleavage. B. Western blot of MDA-MB-231 cells following treatment with doxorubicin indicates cells with reduced PKR have a reduced rate of eIF2α phosphorylation and PARP cleavage. C. Western blot of T-47D cells following treatment with doxorubicin indicates cells with reduced PKR have a reduced rate of eIF2α phosphorylation.

One well-defined mechanism by which PKR can promote apoptosis is by phosphorylation of eIF2α resulting in inhibition of protein synthesis. Therefore, we examined the rate of eIF2α phosphorylation in breast cancer cell lines following treatment with DOX. Significantly, eIF2α phosphorylation is induced in MCF7, T-47D and MDA-MB-231 cells following 4 to 8 hours treatment with 2.5 µM DOX (Figure 6A, B and C; siControl cells). In contrast, breast cancer cell lines with reduced PKR expression by siRNA knockdown have a delayed rate of eIF2α phosphorylation following DOX treatment with significant eIF2α phosphorylation not observed until 24 hours of 2.5 µM DOX treatment (Figure 6A, B and C; siPKR cells). As an indicator of apoptosis, western blotting was also performed for cleaved PARP following times of DOX treatment. Importantly, a significant level of cleaved PARP was observed in MCF7 and MDA-MB-231 siRNA control cells following 16 hours of DOX treatment (Figure 6A and B; siControl cells). In contrast, cleaved PARP was either not observed or greatly reduced in MCF7 and MDA-MB-231 cells with reduced PKR expression by siRNA knockdown (Figure 6A and B; siPKR cells). In addition, cleaved PARP was not observed in either T-47D control or PKR siRNA cells under these conditions (data not shown). Taken together these results suggest that phosphorylation of eIF2α and consequent apoptosis is delayed in cells with reduced PKR compared to control cells following DOX treatment.

**Figure 7:**
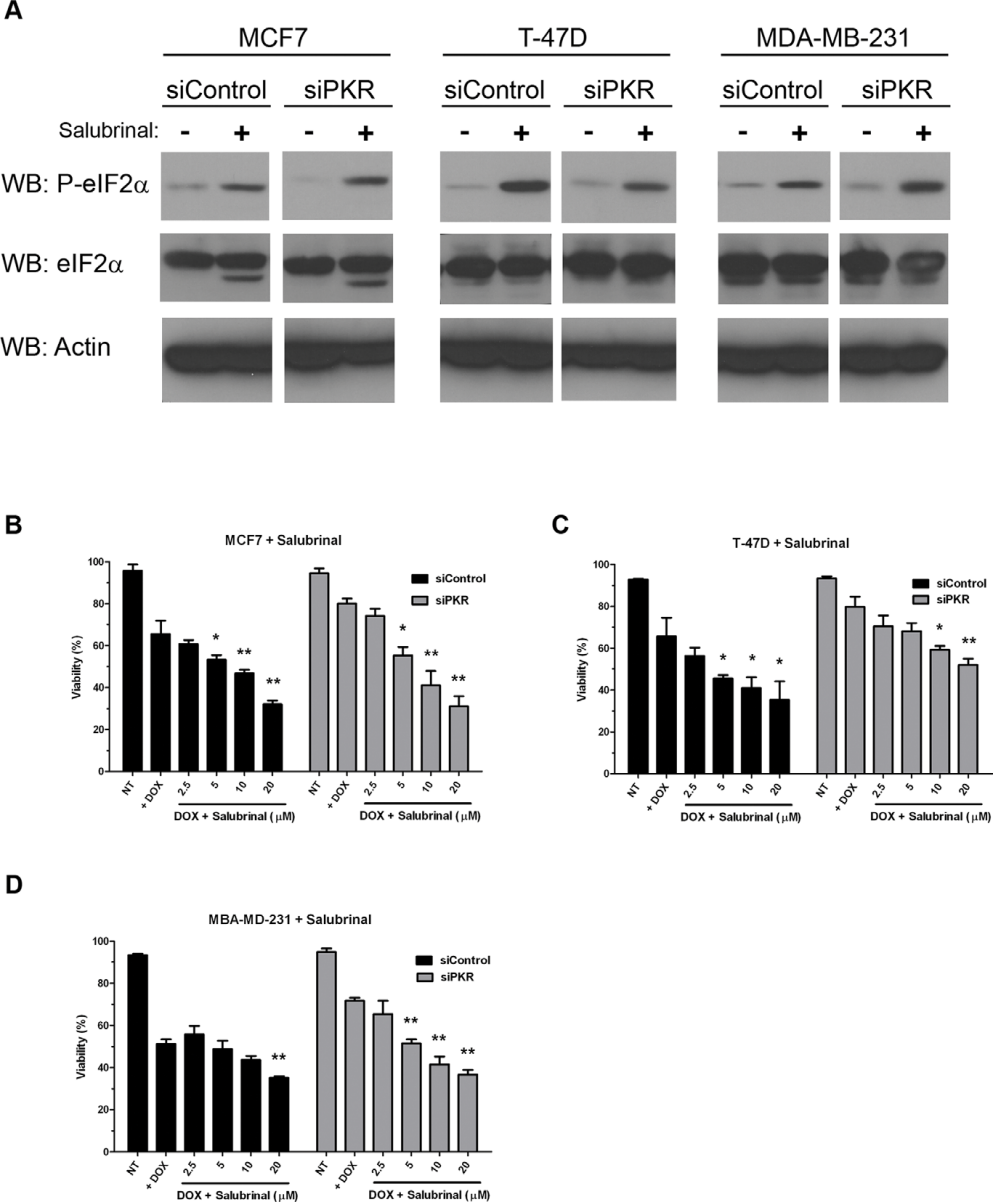
Phosphorylation of eIF2α increases breast cancer cell line sensitivity to doxorubicin. A . Western blotting using antibody specific for phosphoserine-51 eIF2α (P-eIF2α), eIF2α or actin demonstrates increased eIF2α phosphorylation in breast cancer cell lines following 48 hours treatment with 20 µM salubrinal. **B**.–**D**. Breast cancer cell lines either expressing PKR siRNA (siPKR) or control siRNA (siControl) were treated with either 2.5 µM DOX (+ DOX) or co-treated with 2.5 µM DOX and increasing concentrations of salubrinal for 48 hours. Viability was measured by Trypan blue dye exclusion assay. Experiments were repeated in triplicate and mean ± SD graphed. Statistical significance was determined by t-test. * Indicates p<0.05, ** indicates p<0.01 compared to DOX treatment alone.

To further investigate whether induction of eIF2α phosphorylation may promote breast cancer cell sensitivity to DOX, we treated MCF7, T-47D and MDA-MB-231 cells either expressing control or PKR siRNA with salubrinal, a specific inhibitor of eIF2α phosphatase that has been reported to cause an increase in the level of phosphorylated eIF2α [40]. Briefly, MCF7, T-47D or MBA-MD-231 cells were either treated with 2.5 µM DOX alone or co-treated with 2.5 µM DOX and increasing concentrations of salubrinal for 48 hours. Importantly, treatment with 20 µM salubrinal for 48 hours significantly increased the level of phosphorylated eIF2α in all cell lines tested (Figure 7A). Furthermore, co-treatment with salubrinal increased DOX cytotoxicity in both control siRNA and PKR siRNA expressing cell lines (Figure 7B–D). Significantly, salubrinal treatment of cells with reduced PKR restored DOX sensitivity to the level of control cells (Figure 7B–D). Importantly, salubrinal treatment alone did not promote cell death (data not shown). These results indicate that phosphorylation of PKR’s downstream target, eIF2α, is important for the full and potent cytotoxic effect of DOX. Furthermore, treatment with salubrinal may be used to restore DOX sensitivity to cells with reduced PKR expression.

## Discussion

We report that PKR expression is significantly upregulated in primary breast cancer compared to normal or benign breast epithelial tissue. In addition, PKR expression is increased in precancerous stages of mammary cell hyperplasia and dysplasia compared to normal tissues but not cases of breast tissue inflammation. Thus, the oncogenic transformation process itself may play a role in elevated PKR expression similar to what has been observed in colon cancer. [41] Furthermore, and potentially of therapeutic significance, results demonstrate that elevated PKR in breast cancer cell lines may function to specifically enhance doxorubicin (DOX)-induced apoptosis by a mechanism dependent on eIF2α phosphorylation. Significantly, treatment of breast cancer cell lines with salubrinal, to promote eIF2α phosphorylation, augments DOX-induced cell death and suggests that the PKR-eIF2α signaling pathway is important for DOX cytotoxicity in breast cancer. Since DOX has been the backbone of current standard combination chemotherapy regimens for treating breast cancer, we propose that increased PKR in primary breast cancer vs. normal tissue may represent a positive prognostic biomarker for response to chemotherapy and contribute to DOX’s favorable therapeutic index when used to treat breast cancer.

While it is not yet clear how PKR expression can be elevated in primary breast cancer tissues or why increased PKR in such untreated malignancies apparently fails to hinder their cellular growth, we speculate that PKR is in a latent, inactivated state in breast cancer tissue. Thus, upregulation of PKR expression in and of itself may not by sufficient to bring about growth inhibition. Additional selective stresses (i.e. chemotherapy such as doxorubicin) may need to be applied to breast tumor cells to effect PKR activation with inhibition of new protein synthesis and enhanced apoptosis. In support of this, breast cancer cell lines with reduced PKR expression by siRNA knockdown display no change in the rate of cell proliferation under normal growth conditions compared to control cells. Alternatively, PKR’s anti-proliferative function may be suppressed by some additional uncharacterized cellular mechanism(s) such as compensatory changes in gene expression. In support of this, it has been reported that in addition to increased PKR expression, breast cancer cell lines may express increased P58 and eIF2B that could enable these cells to circumvent the growth inhibition effect of increased PKR expression and/or eIF2α phosphorylation. [38] Further studies will be necessary to fully elucidate the features of breast cancer cells that promote increased PKR expression while circumventing PKR’s proapoptotic function.

Breast cancer cell lines with reduced PKR displayed a delay in eIF2α phosphorylation and reduced apoptosis following treatment with DOX. Importantly, treatment with IFN to increase PKR expression or with salubrinal to promote eIF2a phosphorylation restores DOX cytotoxicity in cells with reduced PKR. Furthermore, our data indicate that inhibition of PKR activity with a small molecule compound reduces breast cancer cell sensitivity to DOX. Taken together these results suggest that PKR activity is necessary to obtain the full and potent therapeutic effect of DOX. Thus, future therapeutic approaches that can promote increased expression/activation of PKR and phosphorylation of eIF2α may be an effective modality of treatment for breast cancer patients whose breast tumors do not demonstrate elevated PKR.

## Materials and Methods

### Cell Lines, Antibodies and Other Reagents

MCF10A (lot# 7690599), MCF7 (lot# 7629688), T-47D (lot# 7516238), MDA-MD-231 (lot# 57618051) cells were obtained from ATCC (Manassas, VA). Cells were propagated in Dulbecco modified Eagle medium (DMEM) supplemented with 10% fetal bovine serum (FBS), 1% L-glutamine and 1% Penicillin-Streptomycin in a humidified incubator at 37°C and 5% CO_2_ (Life Technologies, Carlsbad, CA). In addition, medium for MCF7 cells contained 5 µg/ml bovine insulin (Sigma-Aldrich, St. Louis, MO). Doxorubicin (DOX), PKR inhibitor compound (PKRI), paclitaxel and salubrinal were from Calbiochem/EMD Millipore (Darmstadt, Germany). Hydrogen peroxide was from Sigma-Aldrich (St. Louis, MO). Human IFNα was from R&D systems (Minneapolis, MN). Phospho-threonine 451-specific PKR rabbit polyclonal antibody was from Invitrogen/Biosource (Grand Island, NY). PKR M02 monoclonal antibody clone 1D11 was from Abnova (Taipei City, Taiwan). Phospho-serine 51-specific eIF2α, eIF2α and cleaved-PARP antibodies were from Cell Signaling Technology (Beverly, MA). Antibody to actin was from Santa Cruz Biotechnology Inc (Santa Cruz, CA).

### Tissue Microarray IHC Staining and Analysis

Tissue microarrays (TMAs) were obtained from Biomax US (Rockville, MD) and stained by immunohistochemistry (IHC) using monoclonal antibody to PKR. The following arrays were stained: BR722, BR1002, BR1003, BR1006, BR2082, and BN08013. Antibody optimization and staining was performed by the University of Florida’s molecular pathology core facility. Images of the IHC TMA sections were digitized using a Scanscope digital slide scanner and visualized with Imagescope (Aperio, Vista, CA). Three pathologists, blinded to all characteristics of samples, independently quantified PKR immunoreactivity. IHC analysis scores were determined by taking the product of the estimated staining intensity (0 for negative, 1 for weak, 2 for moderate, or 3 for strong) and percentage of tissue stained (0% = 0, <25% = 1, 25%–75% = 2, >75% = 3), giving a range of possible scores between 0 and 9. Scores for replicate cores were averaged to determine a composite score for each case. T-test with F-test was performed using GraphPad Prism version 5 (GraphPad Software, San Diego California USA, www.graphpad.com).

### Knockdown of PKR Expression by siRNA

Transduction-ready lentivirus particles containing shRNAs specific for human PKR were used to knockdown PKR expression in MCF7, T-47D and MDA-MB-231 cells according to the manufacturer’s protocol (Santa Cruz Biotechnology, Inc., #sc-36263). A GFP-expressing control lentivirus was used to measure transduction efficiency and optimize conditions. After transduction, stable cell lines were isolated by selection with 2 µg/ml puromycin. Efficiency of knockdown was evaluated by western blotting.

### Cell Proliferation, Viability and Invasion Measurements

Cell proliferation and viability during normal growth or following treatment either with DOX, H_2_O_2_, or paclitaxel for the indicated concentrations and times were measured by Trypan blue dye exclusion assay. Viable and dead cells were counted with the aid of an Auto T4 Cellometer (Nexcelom Bioscience, Lawrence, MA). In addition, TUNEL assay was performed using an APO-BRDU kit (BD Biosciences, Sane Jose, CA). Cell invasion was measured using a CytoSelect 24-Well Cell invasion assay (Basement membrane, colorimetric) from Cell Biolabs, Inc. (San Diego, CA). Briefly, 20,000 cells were plated in the upper chamber in serum-free medium while medium containing 10% FBS was placed in the lower chamber. After 24 hours, invasive cells were stained and OD 560nm measured according to the manufacturer’s protocol. Statistical significance was calculated by T-test using GraphPad Prism version 5.

## Supporting Information

Figure S1 **Decreased PKR expression in breast cancer cell lines decreases sensitivity to doxorubicin but not paclitaxel. A**. Cells with reduced PKR expression are less sensitive to DOX. Cell viability was measured in MCF7 cells expressing either siRNA specific to PKR (siPKR) or a control siRNA (siControl) at various times following treatment with 5 µM DOX by Trypan blue dye exclusion assay. Experiments were repeated in triplicate and mean ± SD graphed. **B**. TUNEL assay by flow cytometry suggests that MCF7 and T-47D cells expressing PKR siRNA (siPKR) display reduced apoptosis compared to control (siControl) cells after 24 hours treatment with 5 µM DOX. Experiments were repeated in triplicate and mean ± SD graphed. Statistical significance was determined by t-test. * Indicates p<0.05. **C**. Reduced PKR expression does not affect sensitivity to paclitaxel. Viability of MCF7 cells expressing either PKR (siPKR) or control (siControl) siRNA after 24 hours treatment with increasing concentrations of paclitaxel was measured by Trypan blue dye exclusion assay. Experiments were repeated in triplicate and mean ± SD graphed. **D**. Viability after 24 hours co-treatment with 2.5 µM DOX and increasing concentrations of paclitaxel was measured by Trypan blue dye exclusion assay. Experiments were repeated in triplicate and mean ± SD graphed.
(TIF)
