# Supplementary figures and images for: Increased Expression of the dsRNA-Activated Protein Kinase PKR in Breast Cancer Promotes Sensitivity to Doxorubicin
Source: PLoS One. 2012 Sep 24;7(9):e46040. doi: 10.1371/journal.pone.0046040 (PMC3454339; doi:10.1371/journal.pone.0046040)

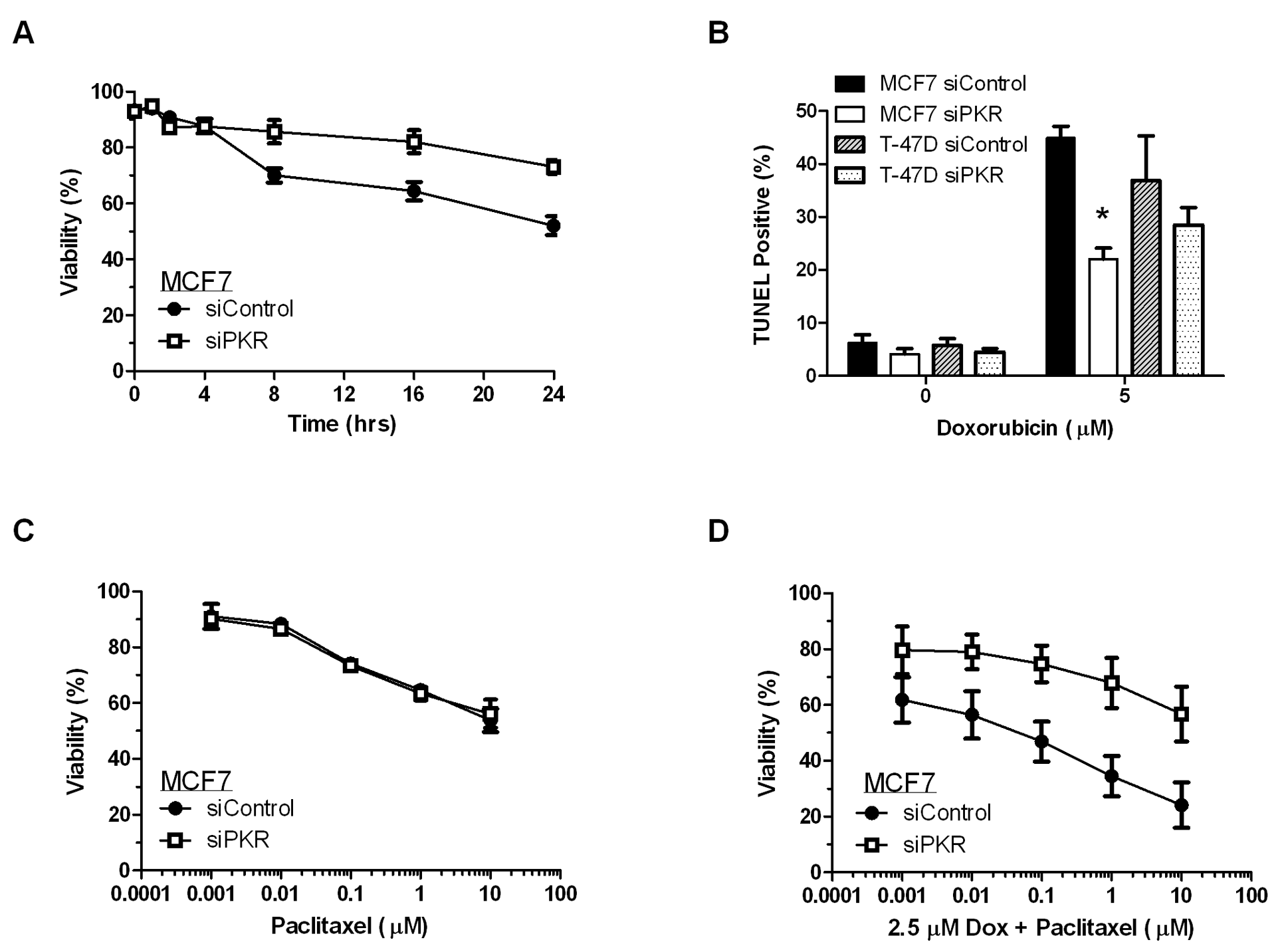

Supplement: Figure S1 — Decreased PKR expression in breast cancer cell lines decreases sensitivity to doxorubicin but not paclitaxel. A. Cells with reduced PKR expression are less sensitive to DOX. Cell viability was measured in MCF7 cells expressing either siRNA specific to PKR (siPKR) or a control siRNA (siControl) at various times following treatment with 5 µM DOX by Trypan blue dye exclusion assay. Experiments were repeated in triplicate and mean ± SD graphed. B. TUNEL assay by flow cytometry suggests that MCF7 and T-47D cells expressing PKR siRNA (siPKR) display reduced apoptosis compared to control (siControl) cells after 24 hours treatment with 5 µM DOX. Experiments were repeated in triplicate and mean ± SD graphed. Statistical significance was determined by t-test. * Indicates p<0.05. C. Reduced PKR expression does not affect sensitivity to paclitaxel. Viability of MCF7 cells expressing either PKR (siPKR) or control (siControl) siRNA after 24 hours treatment with increasing concentrations of paclitaxel was measured by Trypan blue dye exclusion assay. Experiments were repeated in triplicate and mean ± SD graphed. D. Viability after 24 hours co-treatment with 2.5 µM DOX and increasing concentrations of paclitaxel was measured by Trypan blue dye exclusion assay. Experiments were repeated in triplicate and mean ± SD graphed. (TIF) [file pone.0046040.s001.tif]
